# Mechanisms of redox balance and inflammatory response after the use of methylprednisolone in children with multisystem inflammatory syndrome associated with COVID-19

**DOI:** 10.3389/fimmu.2023.1249582

**Published:** 2023-08-14

**Authors:** Stasa Krasic, Vladislav Vukomanovic, Sanja Ninic, Srdjan Pasic, Gordana Samardzija, Nemanja Mitrovic, Maja Cehic, Dejan Nesic, Milica Bajcetic

**Affiliations:** ^1^ Cardiology Department, Mother and Child Health Institute of Serbia, Belgrade, Serbia; ^2^ Faculty of Medicine, University of Belgrade, Belgrade, Serbia; ^3^ Immunology Department, Mother and Child Health Institute of Serbia, Belgrade, Serbia; ^4^ Pathology Department, Mother and Child Health Institute of Serbia, Belgrade, Serbia; ^5^ Faculty of Medicine, Institute of Medical Physiology, University of Belgrade, Belgrade, Serbia; ^6^ Institute of Pharmacology, Clinical Pharmacology and Toxicology, School of Medicine, University of Belgrade, Belgrade, Serbia

**Keywords:** MIS-C, oxidation-reduction potential, superoxide dismutase, catalase, lymphocytes immunophenotype, methylprednisolone

## Abstract

**Background:**

Multisystem inflammatory syndrome in children (MIS-C) associated with being infected with coronavirus-19 (COVID-19) is a life-threatening condition resulting from cytokine storm, increased synthesis of reactive oxygen species (ROSs), and hyperinflammation occurring in genetically predisposed children following an infection with SARS-CoV-2.

**Aim:**

The primary aims of our study were to identify changes in the activity of antioxidant enzymes in erythrocytes and total oxidative status in plasma after being treated with methylprednisolone (MP).

**Methods:**

A prospective cohort study of 67 children (56.7% male) under 18 with MIS-C being treated with MP was conducted at the Mother and Child Health Institute from January 2021 to April 2022. The impact of the therapy was assessed on the basis of the clinical condition, haematological and biochemical blood parameters, and echocardiographic findings.

**Results:**

59.7% of patients presented cardiovascular (CV) manifestations, while myocardial dysfunction was observed in half of all patients (50.7%). A severe clinical course was observed in 22/67 patients. Children with CV involvement had a significantly higher relative concentration of B lymphocytes and lower relative concentration of NK cells than patients without CV issues (p < 0.001 and p = 0.004, respectively). Patients with severe MIS-C had a lower relative count of NK cells than those with moderate MIS-C (p = 0.015). Patients with myocardial dysfunction had a higher total oxidative plasma status (TOPS) than children without (p = 0.05), which implicates pronounced oxidative stress in the former cohort. In patients with shock, lower erythrocytes superoxide dismutase (SOD) activity was observed on admission compared to patients without shock (p = 0.04). After MP was administered, TOPS was significantly reduced, while catalase (CAT) and SOD activity increased significantly. Treatment failure (TF) was observed in 6 patients, only females (p=0.005). These patients were younger (p=0.05) and had lower CAT activity on admission (p=0.04) than patients with favorable treatment responses. In the group of patients with TF, TOPS increased after treatment (before 176.2 ± 10.3 mV, after 199.0 ± 36.7 mV).

**Conclusion:**

MP leads to rapid modulation of TOPS and increases the activity of antioxidant enzymes in erythrocytes resulting in clinical and echocardiographic improvement. Based on the observed changes in the activity of the antioxidant enzymes, we can conclude that s hydrogen peroxide is the dominant ROS in patients with MIS-C. Patients with TF showed reduced CAT activity, whereas the treatment with MP led to pronounced oxidation. This implies that low CAT activity may be a contraindication for using MP.

## Introduction

Multisystem inflammatory syndrome in children (MIS-C) associated with coronavirus-19 (COVID-19) is a life-threatening condition resulting from cytokine storm and hyperinflammation in genetically predisposed children following a symptomatic or asymptomatic infection with SARS-CoV-2. MIS-C manifests 2-6 weeks after the acute COVID-19 condition, and serum SARS-CoV-2 antibodies are detected in most patients (1–8).

Although MIS-C shares some characteristics with toxic shock syndrome (TSS), macrophage activation syndrome (MAS), and Kawasaki disease (KD), the pathophysiological mechanisms of MIS-C are still unknown. The primary pathophysiological mechanism is the uncontrolled activation of the inflammatory cascade that occurs in response to SARS-CoV-2, i.e. as a consequence of the virus-induced autoimmune reaction in genetically susceptible individuals (1, 9).

The virus-induced autoimmune response leads to endothelial dysfunction, increased vascular permeability, capillary leakage, hypoalbuminemia, hyponatremia, hypovolemia, and shock. Hyperinflammation and significant micro-and macrovascular damage lead to an increased synthesis of reactive oxygen species (ROSs) and reactive nitrogen species (RNSs) and to reduced availability and/or increased consumption of antioxidants, disrupting redox homeostasis. The excess of ROSs creates oxidative stress (OS), damaging cellular lipids, proteins, and nucleic acids (10–12).

Previous studies have shown the importance of OS in the acute phase of KD. In patients with KD, administering intravenous immunoglobulins (IVIGs) in the early phase reduces the ROS level and inflammatory response by an independent mechanism; therefore, ROS levels might be a valuable biomarker for evaluating the response to therapy (13–15). Although no prospective studies have investigated OS in MIS-C, and the OS was modulated after the therapy, we believe that OS has a significant pathophysiological role due to its similarity with KD.

The primary study aims were to (1) identify changes in the activity of antioxidant enzymes in erythrocytes following treatment with methylprednisolone (MP) and indirectly assess the activity of dominant reactive species in children with MIS-C associated with COVID-19 (2); identify changes in total oxidative plasma status (TOPS) following treatment with MP (3); evaluate the inflammatory response by determining the peripheral blood immunophenotype. The secondary aim was to determine the correlation between OS parameters and the peripheral blood immunophenotype using biochemical and haematological blood parameters and echocardiographic findings.

## Methods

**Figure 1:**
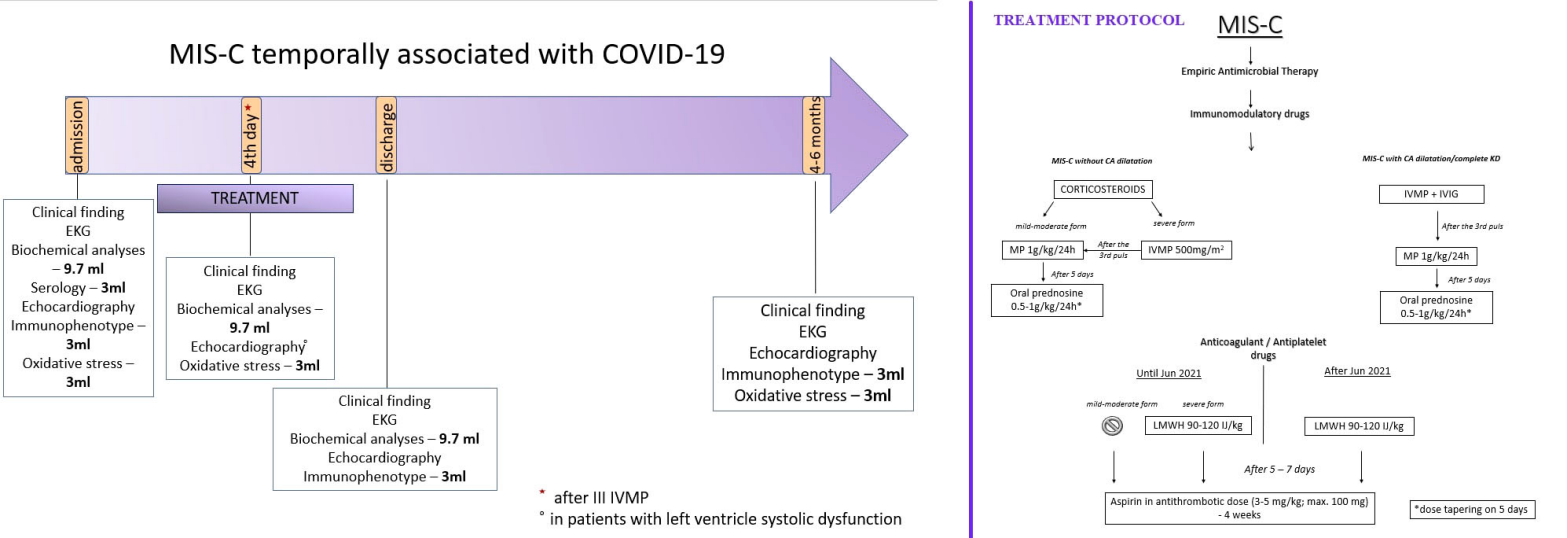
The manuscript methodology graphical presentation with our Institutional treatment protocol.

A prospective cohort study of children under 18 with MIS-C associated with COVID-19 was conducted at the Mother and Child Health Institute from January 2021 to April 2022. The MIS-C diagnosis was made according to the WHO’s recommendations (4). Patient evaluation on admission involved a detailed medical history, complete physical examination, and standard haematological and biochemical blood analyses. Blood samples were also taken to determine OS parameters, the SARS-CoV-2 antibody level, and the peripheral blood immunophenotype (**Figure 1**). On admission, an ECG was taken, and an echocardiographic examination was performed. Patients were treated with corticosteroids (CSs) in the form of pulses of MP or MP in standard doses. The treatment protocol is shown in **Figure 1**.

The impact of the therapy was assessed based on the clinical condition, haematological and biochemical blood parameters, and echocardiographic findings. On the third day in the hospital, the following was evaluated: standard haematological and biochemical blood analyses, oxidation-reduction potential, and activity of antioxidant enzymes in erythrocytes. An echocardiographic examination was also performed if the patients had signs of myocardial dysfunction on admission.

Before discharge, standard haematological and biochemical blood analyses, the immunophenotype of peripheral blood lymphocytes, and the echocardiographic examination were re-examined.

The clinical condition, laboratory analyses, redox status, immunophenotype of peripheral blood lymphocytes, and echocardiographic findings were evaluated 4-6 months after the acute illness.

Treatment failure (TF) was defined as the persistence of fever (> 38°C or > 100.4°F) for 48 hours after therapy initiation or the occurrence of acute left ventricular (LV) systolic dysfunction (ejection fraction (EF) < 55%) and a need for vasoactive drugs.

### Inclusion criteria

Patients with MIS-C associated with COVID-19 being treated at the Mother and Child Health Institute between January 2021 and April 2022 were included in the study. The diagnosis was made based on the WHO’s recommendations (4):

1) Fever and elevated inflammatory markers for 3 days or more,
2) 2 of the following: (A) rash or bilateral non-purulent conjunctivitis or mucocutaneous inflammation signs (oral, hands, or feet); (B) hypotension or shock; (C) features of myocardial dysfunction, pericarditis, valvulitis, or coronary abnormalities (including echocardiogram findings or elevated troponin or N-terminal pro-B-type natriuretic peptide); (D) evidence of coagulopathy (elevated prothrombin time, partial thromboplastin time, and elevated D-dimers); and (E) acute gastrointestinal problems (diarrhoea, vomiting, or abdominal pain),
3) Positive RT-PCR, antigen test, or serology; or any contact with patients with COVID-19,
4) Exclusion: other microbial cause of inflammation.

### Exclusion criteria

All children who (1) used antioxidants (2), had elevated oxidative stress: smoking, congenital metabolic diseases, or (3) had chronic kidney disease, systemic or pulmonary hypertension, or endocrinological disorders were excluded from the study.

### Ethics statement

Informed written consent was signed by the parents of all children, along with assent where appropriate. Informed consent was given after the nature, and possible consequences of the studies were explained. The Institutional Ethics Committee approved the study (No 8/62).

### Determination of oxidative stress parameters

Sample preparation and analysis of antioxidant enzyme activity and oxidation-reduction potential (ORP) were performed at the Institute of Pharmacology, Clinical Pharmacology and Toxicology, Faculty of Medicine in Belgrade.

### Activities of erythrocytes antioxidant enzymes

From all subjects, 3 ml of peripheral venous blood was collected with a heparinized syringe (0.2 ml of heparin). Erythrocytes and plasma are separated by centrifugation (10 minutes at 5000 rpm, 4°C). Separated erythrocytes are washed three times with physiological solution by centrifugation (10 minutes at 5000 rpm, 4°C), and thus prepared are stored at -80°C. Before the start of the work, the samples are thawed, and the activity of the enzymes superoxide dismutase (SOD), catalase (CAT), glutathione peroxidase (GSH-Px), glutathione reductase (GR) is determined in them by the spectrophotometry method on the HEλIOS device (Thermo Spectronic, UK).

Aliquots of three times washed erythrocytes were lysed by ice-cold distilled water. Hemoglobin (Hb) concentration was measured by the method of Drabkin and Austin (16). To determine the activity of Cu/Zn SOD, it was necessary to remove hemoglobin by Tsuchihashi (17). The activity of erythrocyte Cu/Zn SOD was measured according to the Misra and Fridovich method, catalase (CAT) according to the Beutler, glutathione reductase (GR) according to the Glatzle, and glutathione peroxidase (GSH-Px) according to Paglia and Valentine method (18–21).

### Oxidation-reduction potential (ORP)

TOPS was the static measure of ORP which was determined at room temperature on a RedoxSYS System (AytuBioScience, Inc., Englewood, CO). The plasma (40 µL) sample is dripped onto the previously placed sensor, after which the automatic reading process begins. After 5 seconds, when the ORP values ​​become stable, the millivolts (mV) value is read. Higher TOPS, i.e. static ORP values, stand for pronounced oxidation (22).

### Flow cytometry

3 ml of whole blood samples in a tube with EDTA were submitted to the Immunological Laboratory of the Mothers and Children Health Institute of Serbia for lymphocyte immunophenotyping.

100 μl of whole blood was mixed with 20 µl of Multitest™ 6-Color in 1 TBNK tube and the second HU TH1/2/17 Phenotyping Kit. The test tubes were incubated at room temperature, in the dark, for 15 minutes and lysed with FACS lysis buffer (Becton Dickinson) for 15 minutes. Before staining with the HU TH1/2/17 Phenotyping Kit, the sample was incubated with an activation cocktail (Leukocyte Activation Cocktail, with BD GolgiPlug) according to the manufacturer’s recommendations. After centrifugation and subsequent washing, the cells were suspended in 400 µl of cell-wash solution (Becton Dickinson) and analyzed using the FACS CANTOII cytometer (Becton Dickinson) in the DIVA software.

### Statistics

The descriptive statistics used included mean, median, standard deviation (SD), interquartile range (IQR), and the total number and percentage (%) of monitored parameters. The difference in the distribution of specific parameters among the studied groups was determined using χ2 or Fisher’s test. Shapiro–Wilk and Kolmogorov–Smirnov tests were used to test the normality of the distribution of numerical variables. Groups were compared using Student’s t-test and the Mann–Whitney test. Paired t-tests and the Wilcoxon test were used to compare 2 related samples. Pearson or Spearman tests were tested for correlation between parameters in different groups. The data were processed using the statistical software SPSS 25.0 for Windows 10. All statistical methods were considered statistically significant, p ≤ 0.05

Using a sample size calculator for a significance level (CI) of 95% and a margin of error of 5%, the estimated sample size is 23 patients.

## Results

The study included 67 patients, 38 male (56.7%) and 29 female (43.3%); the mean age was 9.2 (IQR 6.3–12.9) years. There was no difference in age between genders (p = 0.2). Serological blood tests for SARS-CoV-2 were positive in 64 patients (95.5%), while 3 (4.5%) had positive PCR or Ag tests from a nasopharyngeal swab.

All patients had a fever lasting a median of 5 (IQR 4-6) days. Most children (75%) had gastrointestinal manifestations. Three patients underwent appendectomies. Hepatitis and pancreatitis were present in 18/67 and 7/67 patients respectively. Cardiovascular manifestation was present in 40/67 patients (59.7%), while myocardial dysfunction was observed in half of all patients (50.7%). Children with myocardial dysfunction were older than patients with normal myocardial function (10.2 (IQR 7.7 – 13.9) vs 7.5 (4.2 – 11.6) years; p = 0.04). A severe clinical course and ICU admission were observed in 22/67 patients. These patients more often had hepatitis (p = 0.003) and pancreatitis (p = 0.004). A 3-year-old boy with moderate MIS-C had intracardiac thrombosis. A 7-year-old patient, who had previously undergone cardiac surgery to correct transposition of the great arteries in the neonatal period, died, on the third day in hospital.

Initial laboratory findings revealed elevated proinflammatory markers (CRP, fibrinogen, D-dimers) and low serum sodium, phosphate, albumin, and platelet counts (**Table 1**). Elevated cardiac troponin I (cTnI) and proBNP were observed in 44.9% and 83.9% of patients. The average proBNP was 2455.5 pg/mL (IQR 1352.7–>5000). In patients with elevated cTnI, the median cTnI value was 0.27 ng/mL (IQR 0.17–0.59).

**Table 1:** Difference between laboratory analysis at admission, 3rd in hospital day, and at discharge.

Laboratory analysis	Admission	3rd day	discharge	P-value Admission-3rd day Admission-discharge
C-reactive protein (mg/L)	127.8 (92.7-206.8)	50.2 (27.5 – 96)	3.1 (1.4 – 7.3)	<0.001 <0.001
Platelet count (* 10^9^)	152 (111.7 – 206.2)	215 (146 – 326)	505 (417 – 640)	<0.001 <0.001
Sodium (mmol/L)	133 (130 – 135)	137 (136 – 139)	137 (136 – 139)	<0.001 <0.001
Albumin (g/L)	35 (31 – 38)	33 (30 – 36)	39 (37 – 41)	0.08 <0.001
Phosphate (mmol/L)	1 (0.85 – 1.17)	1.04 (0.84 – 1.23)	1.29 (1.15 – 1.46)	0.388 <0.001
LDH (IU/L)	546 (473 – 643)	421 (386.2 – 544.5)	434 (363 – 486)	<0.001 <0.001
SGOT (IU/L)	31 (21 – 52)	19.5 (14.2 – 30.5)	21.0 (17.0 – 34.5)	<0.001 <0.001
SGPT (IU/L)	24 (17 – 48)	24 (15 – 48.5)	36 (21 – 61.2)	0.19 0.03
Fibrinogen (g/L)	5.4 (4.1 – 6.6)	3.1 (2.45 – 23.75)	2.4 (2 – 2.8)	0.002 <0.001
D – dimers (ng/mL)	739 (397 – 1445)	549 (327 – 725)	207 (127 – 416)	0.08 <0.001
proBNP (pg/mL)	1907.5 (977.7 – >5000)	2252 (553.9 – 3858.7)	153.0 (86.2 – 292.5)	0.12 <0.001
cTnI (ng/mL)	0.1 (0.05 – 0.25)	0.17 (0.1 – 0.4)	0.1 (0.08 – 0.13)	0.183 0.168
LV EF (%)	58 (52 – 68)	59 (56 – 65)	69 (65 – 73)	0.007 <0.001
LV EDD Z score	0.25 (-0.6 – 0.9)	0.5 (-0.2 – 1.22)	-0.1 (-0.8 – 0.5)	0.4 0.003

LDH, lactate dehydrogenase; SGOT, serum glutamic-oxaloacetic transaminase; SGPT, serum glutamic-pyruvic transaminase; cTnI, cardiac troponin I; proBNP, pro B type natriuretic peptide.

Patients’ age correlated positively with CRP and fibrinogen on admission (rr = 0.38, p = 0.002; rr = 0.34, p = 0.007, respectively). CRP correlated positively with fibrinogen (rr = 0.35, p = 0.01). The cTnI level on admission correlated positively with proBNP (rr = 0.4, p = 0.009). A moderate positive correlation was found between proBNP and the relative count of B lymphocytes (rr = 0.54, p = 0.007), whereas proBNP correlated negatively with NK cells (rr = -0.58, p = 0.003). A mild to moderate negative correlation was found between proBNP and albumin (rr = -0.3, p = 0.03) and between proBNP and sodium level (rr = -0.27, p = 0.04). CRP and proBNP correlated mildly to moderately positively (rr = 0.31, p = 0.02). A moderate negative correlation was found between platelet count and D-dimers (rr = -0.4, p < 0.001).

In patients with CV manifestations, higher serum concentrations of CRP and proBNP were observed in comparison to patients without CV manifestations (CRP 186.5, IQR 117.3–253.7, vs 101.8, IQR 54.2–134.1 mg/L, p < 0.001; proBNP 3418, IQR 1576.0–5750.0 vs 938, IQR 406.5–1821, p < 0.001). Children with CV involvement had a significantly higher relative concentration of B lymphocytes and lower relative concentration of NK cells than patients without CV manifestations (B cells 35.5, IQR 25.5–46.2 vs 20.5, IQR 12.7–23.2%, p < 0.001; NK cells 9.0, IQR 5.75–14.0 vs 15.5, IQR 13.7–19.0%, p = 0.004). Patients with severe MIS-C had a lower relative count of NK cells than those with moderate MIS-C (9, IQR 4.5–13.5% vs 14.0, IQR 12.0–17.0%; p = 0.015).

**Figure 2:**
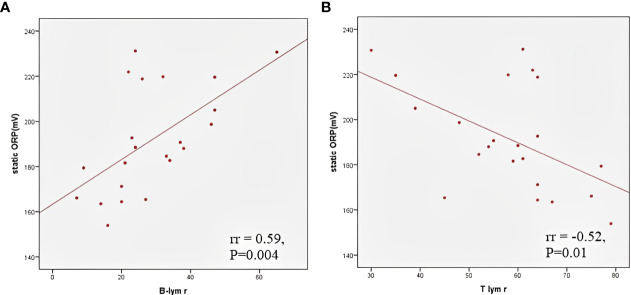
Correlation between total oxidation-reduction stress potential and relative B **(A)** and T **(B)** lymphocyte count.

The TOPS and the values ​​of individual antioxidant enzymes on admission are shown in **Table 2**. The CAT level was higher in male patients than in female patients (129668.7 ± 20641.4 vs 96203.8 ± 36893.9μmol H_2_O_2_/min/g Hb; P=0.006), while girls had a higher GSH-Px level than boys (22.1 ± 4.7 vs 17.1 ± 3.1 μmol NADPH/min/mg Hb; P=0.004). Patients with myocardial dysfunction had a higher TOPS than children without myocardial involvement (191.7, IQR 184.1 – 219.0 vs 171.2, IQR 165.4 – 188.0 mV; P=0.05). A moderate positive correlation was found between TOPS and proBNP (rr = 0.49, p = 0.01), TOPS and CRP (rr = 0.4, p = 0.04), and TOPS and relative concentration of B lymphocytes (rr = 0.59, p = 0.004), while a negative correlation was observed between TOPS and relative concentration of T lymphocytes (rr = -0.52, p = 0.01) (**Figure 2**). In patients with shock, lower erythrocyte SOD activity was observed on admission in comparison to patients without shock (2002.8 ± 255.6 vs 2269.8 ± 341.5 U/g of Hb; P=0.04), and to patients admitted to the ICU (2020.7 ± 231.5 vs 2292.1 ± 355.9 U/g of Hb; P=0.04).

**Table 2:** Total oxidation-reduction potential, activities of antioxidant enzymes from erythrocytes, and lymphocyte immunophenotype before and after methylprednisolone therapy.

	Before treatment	After treatment	P-value	Follow-up
Static ORP(mV)	188 (IQR 169.9 – 211.9)	176.3 (IQR 161.3 – 187.6)	**0.04**	176.7 (IQR160.2 – 200.6)
CAT (μmol H_2_O_2_/min/gHb)	121715 (IQR 99409.5 – 138218.8)	137833.6 (IQR 122799.1 – 148845.9)	**0.028**	150627 (IQR 138129.1 – 157628.7)
GSH –Px (μmol NADPH/min/mg Hb)	17.9 (IQR 15.2 – 22.1)	17.6 (IQR 15.3 – 20.4)	0.92	12.7 (IQR 10.5 – 15.2)
GR (μmol NADPH/min/mg Hb)	4.8 (IQR 4.4 – 5.6)	4.7 (IQR 4.3 – 5.5)	0.96	4.8 (IQR 4.14 – 5.6)
SOD (U/g of Hb)	2160.1 (IQR 1868.1 – 2507.9)	2665.5 (IQR 2369.5 – 3035.5)	**0.002**	2709.6 (IQR2528.1 – 3135.4)
B-lym(%)	25 (IQR 20.2 – 37.7)	31.5 (IQR 22.2 – 39.7)	0.97	13.5 (IQR 10.2 – 17.5)
T-lym(%)	59.5 (IQR 50.5 – 64.0)	61 (IQR 50.7 – 71.0)	0.12	71 (IQR 68 – 74.5)
CD4/CD3+ (%)	32.5 (IQR 23.7 – 38.0)	30.5 (IQR 26.5 – 34.5)	0.39	35.5 (IQR 31.7 – 37.0)
CD8/CD3+	21.0 (IQR 14.7 – 24.0)	25.0 (IQR 18.2 – 28.5)	0.10	26 (IQR 23.2 – 33.0)
NK cells	13.5 (IQR 7.25 – 15.0)	6.5 (IQR 4.0 – 12.7)	0.08	13 (IQR 11 – 19.25)

ORP, oxidation-reduction potential; Gp-x, glutathione peroxidase; GR, glutathione reductase; SOD, superoxide dismutase.

The bold values have statistically significance.

**Figure 3:**
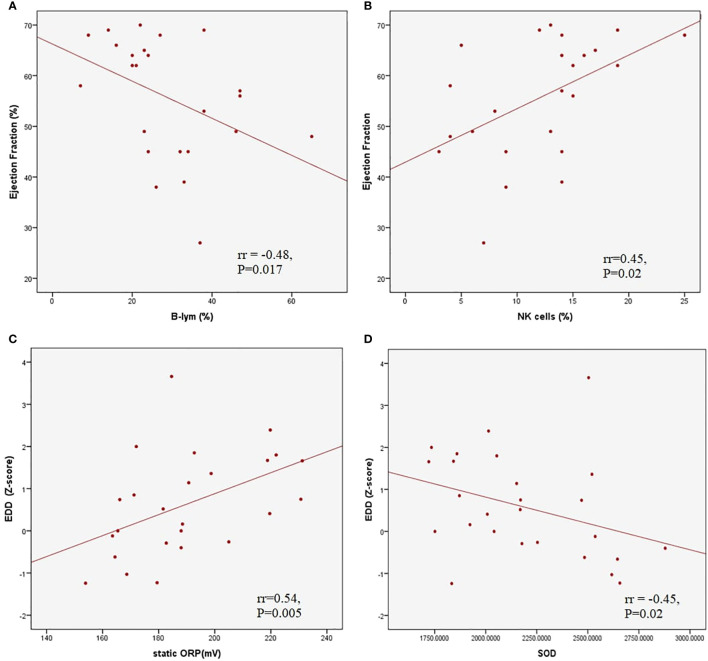
Correlations between left ventricle ejection fraction and relative B **(A)** and Natural Killer **(B)** lymphocyte count; correlations between left ventricle end diastolic dimater Z score and total oxidation-reduction potential **(C)** and superoxide dysmutase activity **(D)**.

The average LV EF was 50.7 ± 13.2% in patients with myocardial dysfunction. A moderate negative correlation was found between LV EF and the relative count of B lymphocytes (rr = -0.48, p = 0.017) and a positive correlation between LV EF and relative NK cells (rr = 0.45, p = 0.02). The left ventricle end-diastolic diameter (EDD) Z score correlated positively with TOPS (rr = 0.54, p = 0.005) and negatively with SOD activity (rr = -0.45, P=0.02) (**Figure 3**). A moderate negative correlation was observed between LV EF and proBNP (rr = -0.58, p < 0.001), and LV EF and CRP (rr = -0.41, p = 0.001).

### Treatment

All patients were treated with MP; 79.1% received pulses of MP. Blood samples were taken from all patients after the MP treatment. TOPS decreased significantly, while CAT and SOD increased after MP administration (**Table 2**). On the other hand, CRP, fibrinogen, D-dimers, and proBNP decreased, while sodium, phosphate, and albumin increased significantly (**Table 1**). After the therapy, significant improvement in LV EF was observed as soon as the third day in the hospital (p = 0.007), and a significant reduction in LV EDD was observed on discharge (p = 0.003).

**Figure 4:**
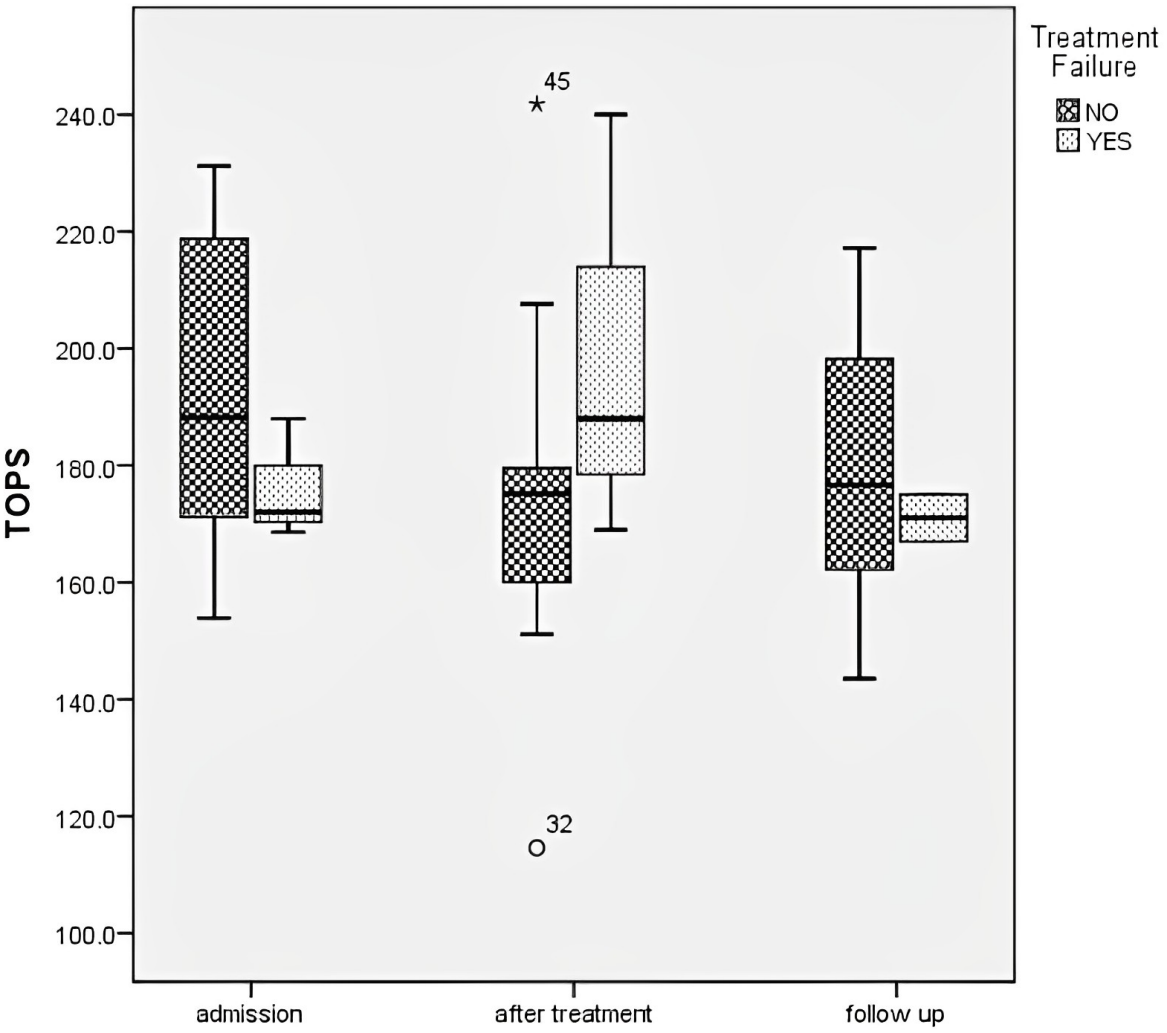
Total oxidation-reduction potential dynamics in different groups regarding treatment failure.

TF was observed in 6 patients; all were treated with MP pulses. Only females had TF (P=0.005). These patients were younger than patients with favorable treatment responses (5.8 (IQR 3.3 – 8) vs 9.5 (IQR 6.6 – 13.1) years; P=0.05). These patients had lower CAT activity on admission than those without TF (98942, IQR 79337 - 102569 vs 123378, IQR 109669 – 141778.9 μmol H_2_O_2_/min/g Hb; P=0.04). Additionally, patients with CAT and SOD activity lower than 117000μmol H_2_O_2_/min/g Hb and 1800U/g of Hb, respectively, had more frequent TF (p=0.03 and p=0.027, respectively). In the group of patients with TF, no TOPS reduction or CRP were noted (p = 0.42, p = 0.16, respectively). In those patients, TOPS increased after treatment (before 176.2 ± 10.3 mV, after 199.0 ± 36.7 mV) (**Figure 4**). The severity of the clinical presentation did not affect the outcome of the disease and TF.

14 patients received inotropic drug support–dopamine with/without dobutamine for 3 days (IQR 2.5–4.5). In patients treated with dopamine, SOD activity was significantly lower on admission (2020.66 ± 231.5 vs 2292.0 ± 355.9 U/g of Hb; p = 0.04).

### Follow-up

**Figure 5:**
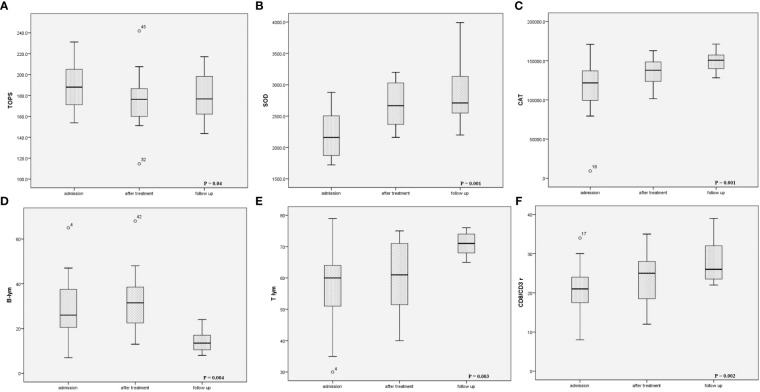
Changes of **(A)** oxidation-reduction potential, **(B)** superoxide dismutase activity, **(C)** catalase activity, **(D)** B-lymphocytes relative count, **(E)** T-lymphocytes relative count, and **(F)** CD8/CD3+ during follow up period.

Control laboratory analysis was refused by 5.9% of patients’ parents (4 patients loss to follow-up), while echocardiography was performed in all children. Control laboratory analysis six months after acute illness yielded entirely normalized results (**Table 2**). Echocardiography examination identified normal systolic function of the LV (EF 66.4 ± 4.1%) and EDD (Z score 0.12, IQR -0.57–0.59). A significant decrease in the relative count of B lymphocytes was found compared to the relative count of B lymphocytes during acute illness (P = 0.004), while T lymphocytes increased significantly (p = 0.003). In patients with CV manifestations, significant elevation in the relative count of NKs cells was observed in the follow-up period compared to on admission (13, IQR 11–22% vs 9, IQR 5.8–14%; p = 0.04). Reduction of TOPS was observed in comparison to the TOPS on admission (p = 0.04), while SOD and CAT increased significantly (p = 0.001) (**Figure 5**).

## Discussion

The primary pathophysiological mechanism of MIS-C is the uncontrolled activation of the inflammatory cascade in response to SARS-CoV-2 (10).

Clinical presentation in MIS-C patients varies and involves a systemic cytokine storm (IL6, CCL2, CXCL8, CXCL9, CXCL10, CXCL11) with gastrointestinal, cardiac, vascular, hematologic, mucocutaneous, neurologic, and/or respiratory pathology and fever (23). Half of our patients had myocardial dysfunction, while almost 20% had shock. Myocardial dysfunction was commonly observed in older children. The mortality rate is around 2% (23), but in our study, only 1 patient, who had undergone surgery to correct transposition of the great arteries, died.

Endothelial dysfunction and hyperinflammation increased vascular permeability and decreased sodium, albumin, and phosphate levels, while elevating CRP, fibrinogen, D-dimers, and proBNP. We found a negative correlation between proBNP and sodium and albumin level. In contrast, proBNP correlated positively with CRP. These laboratory findings can be explained by highly intensive inflammation resulting in third-space loss of fluid, sodium, and albumin or renal sodium loss and myocardial dysfunction.

Oxidative has also been suggested as a major mechanism that causes endothelial dysfunction and primary pathophysiological processes in autoimmune diseases, KD, and TSS (10, 11). Additionally, we found that TOPS on admission correlated positively with CRP and proBNP, which means patients with much more intense inflammation had higher TOPS and more pronounced myocardial dysfunction. ROSs can directly impair contractile function by modifying proteins central to excitation-contraction coupling (24), negatively affect the disposition of myocardial calcium (Ca^2+^), cause arrhythmia, and contribute to cardiac remodeling by inducing hypertrophic signaling, fibrosis, apoptosis, and necrosis (25). Consequently, myocardial stunning in MIS-C might progress to myocardial necrosis. Authors have previously found a relationship between the severity of OS and the New York Heart Association (NYHA) functional class, hs-CRP levels and proBNP in patients with heart failure (26).

Significant endothelial damage and increased cytokine concentration results in an increase in NADPH oxidase and myeloperoxidase (MPO) activity, leading to further “leakage” of superoxide anions (O_2_^−^) into the extracellular space and increased consumption of antioxidants, resulting in OS. O_2_^−^, the most abundant radical species, is also the first stage of the bacterial-killing reaction, which is followed by the production of other free radicals, such as hydrogen peroxide (H_2_O_2_) by SOD (27). We showed that our patients had low SOD levels before MP treatment, especially those admitted to the ICU. This is in line with evidence that in patients with a diagnosis of hyperinflammatory syndrome, including MIS-C, there are increases in lipoperoxidation as well as reduced antioxidant capacity (10, 28). Additionally, SOD activity correlated negatively with LV EDD Z score, which can be explained by the fact that O_2_^−^ shows a concentration-dependent negative inotropic effect (29). Abnormal RyR2 function caused by OS leads to diastolic Ca^2+^ leaking and depletes sarcoplasmic reticulum Ca^2+^ stores. It reduces cytoplasmic Ca^2+^ transients, impairing contractile force generation (30).

Coronary artery involvement is more frequent in KD than in MIS-C, with MIS-C patients instead having myocardial stunning, which can be explained by the different cytokine profiles. In KD, elevated IL17A suggests more pronounced arterial damage in KD than in MIS-C, while chemokines lead to myocardial dysfunction (31, 32).

Our patients had elevated B cells and decreased T cells. Additionally, as the total OS increased, the B lymphocyte level increased, and the T cells decreased. Previous studies have shown an increase in absolute numbers of naïve B cells (short-lived plasmablasts), immature B cells, and atypical memory B cells, all of which fit with a potential humoral response in patients with MIS-C, often weeks after clearance of SARS-CoV-2, raising the possibility that these are autoreactive expansions of antibody-secreting cells (25, 33). Patients with CV involvement had higher levels of B cells, which can be explained by the fact that patients with a severe form of MIS-C formed autoantibodies that bind to endothelial cells, contributing to endothelial dysfunction and multisystemic inflammation, which is characteristic of these patients (10, 20, 34, 35). In our MIS C patients before MP therapy, a decrease in LV EF was noted with an increase in the relative concentration of B cells and raised proBNP.

We showed that NK cells were lower in patients with CV involvement. A positive correlation was found between LV EF and NK cells on admission. Patients with myocarditis, dilatative cardiomyopathy (DCM), and coronary artery disease have severely low NK cells and NK cytotoxicity levels, leading to defects in their frequency and functionality. Although myocardial dysfunction in MIS-C has not been shown to be the consequence of direct myocardial injury but rather immune system-mediated myocardial stunning, it has been concluded that NK cells may be able to directly control autoimmune inflammation of the heart, such as in myocarditis or DCM (36, 37).

We have previously shown that CSs are the best choice of drug in patients with MIS-C associated with COVID-19 because they lead to prompt normalization of body temperature, biochemical and haematological blood parameters, and echocardiographic parameters (37, 38).

**Figure 6:**
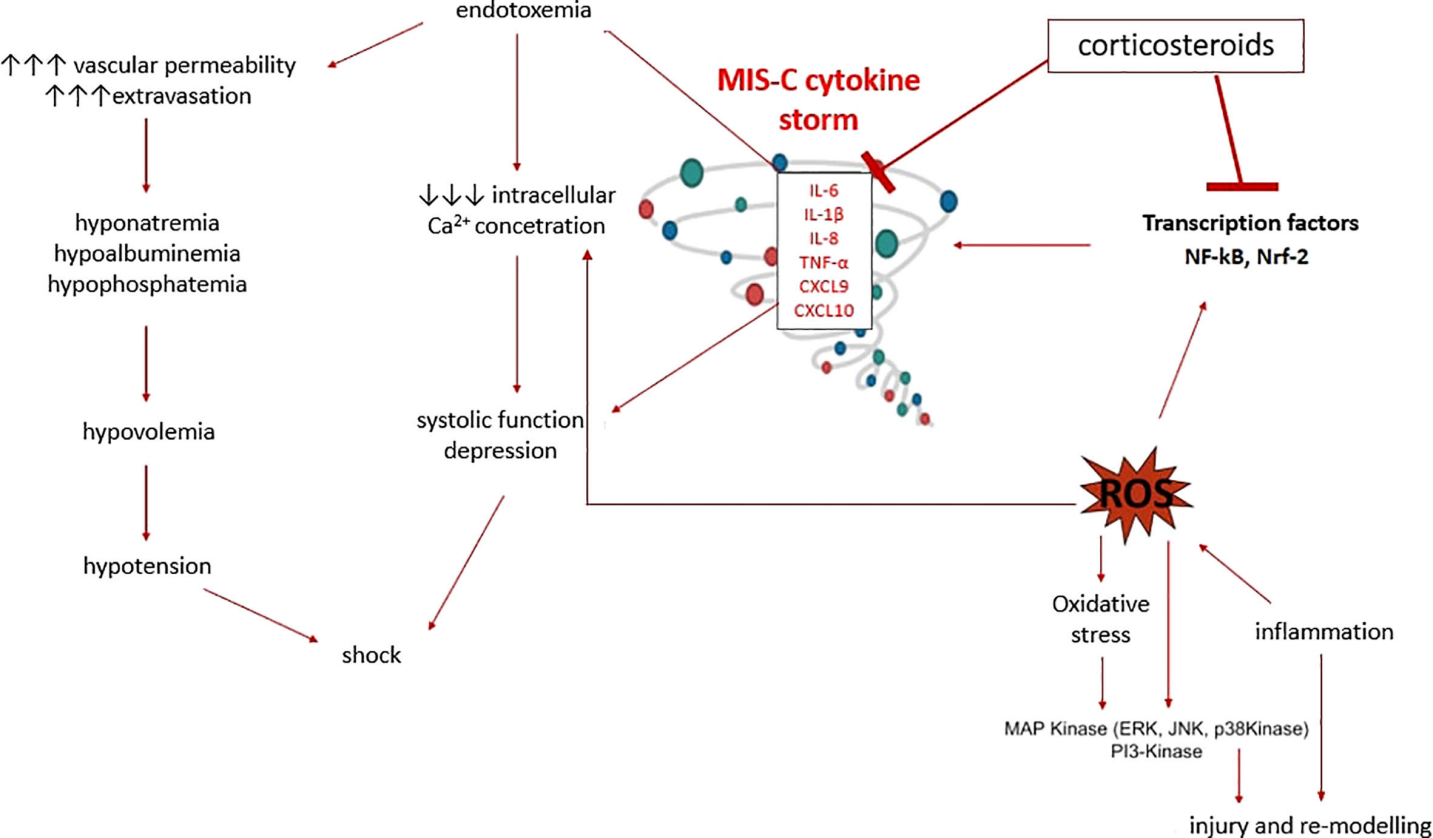
MIS-C pathogenesis and corticosteroids effects.

Through their genomic effects, CSs reduce promoter activities on pro-inflammatory genes and increase the expression of anti-inflammatory mediators. Another mechanism of action of CSs is transrepression, which leads to suppressed expression of immunoregulatory and proinflammatory proteins such as cytokines (IL1, IL2, IL6, TNF-α, IFN-γ) and prostaglandins. The rapid non-genomic effects of CSs also play an essential role, as clinical effects can be observed quickly after administration of high doses, because they promptly reduce the hyperinflammatory response, suppressing vasodilation and increasing vascular permeability by inhibiting the expression of cytokines (TNF-α, IL-6, IL -1α, IL-1β and chemokines: CXCL9 and CXCL10) within a few minutes (**Figure 6**). IVIGs decrease IL6 concentration on the third and fourth day of the disease, and the level of CXCL9 and CXCL10 decreases after only 5 days (37–39), which could explain the better response to CSs in MIS-C patients (29).

In patients with KD, IVIGs in the early phase reduce the ROS level and the inflammatory response by an independent mechanism (13–15). In our study, in MP-responding patients, the ROS concentration significantly decreased immediately after the end of the treatment. TOPS may therefore be a useful biomarker when assessing response to therapy. Additionally, we showed that CAT and SOD activity increased after therapy.

Treatment failure was observed only in the female population and in younger patients. On admission, patients with TF had CAT and SOD activity lower than 117000 μmol H_2_O_2_/min/g of Hb and 1800U/g of Hb, respectively. Therefore, the combined effect of O_2_^−^and H_2_O_2_plays a significant role in the TF. In these patients, besides the treatment, TOPS increased.

During the follow-up period, we showed that the relative count of T cells increased, with B cells decreasing in observed patients. Rajamanickam A et al. concluded that 6–9 months post-recovery, the numbers of naïve, immature, and atypical memory B cells were reduced. In contrast, the numbers of classical memory, activated memory, and plasma cells were increased compared to pre-treatment numbers (33). Additionally, the NK cell level increased in patients with CV manifestation during the follow-up period of our investigation. In patients with coronary artery disease, a 12-month follow-up showed that a continued deficiency of NK cells was correlated with low-grade cardiac inflammation. In contrast, patients that had restored circulating NK cells had little to no cardiac inflammation (36). The activity of both enzymes, SOD and CAT, were suppressed at the admission, in the acute phase of illness, before MP therapy. The reduction of hyperinflammation in our study was accompanied by restoring the activity of the SOD and CAT, the enzymes of antioxidant defense, during the 4 to 6 months follow-up period.

### Limitations

The most significant limitation of the study is the limited number of patients, which makes it difficult to derive definitive statements about pathogenesis and redox balance modulation after the therapy was applied. Randomized multicentre studies need to be conducted in order to establish treatment protocols with a significant level of recommendation.

## Conclusion

The etiopathogenesis of and best treatment for MIS-C associated with COVID-19 are still unknown, but it is a life-threatening condition which other viruses can probably provoke. Our study showed that humoral immune cells play a significant role in the pathogenesis of MIS-C, while patients with a low concentration of NK cells develop myocardial dysfunction. In the acute phase, ROS are elevated, and the dominant ROS is hydrogen peroxide. Erythrocytes serve as a sink for hydrogen peroxide in the circulation and, in this way, protect blood vessels. Patients with significantly greater inflammation had higher TOPS and proBNP with myocardial dysfunction. We have previously suggested MP as the best-choice therapy for MIS-C. The use of MP leads to a rapid modulation of TOPS, which correlates with an improvement in the clinical condition and a decrease in the concentration of positive reactants (CRP, fibrinogen) of the acute phase and an increase in negative reactants (albumin). MP treatment led to a rapid increase in antioxidant defence enzymes. TF was noted in younger children and girls only. These patients had significantly lower CAT values ​​on admission, meaning that hydrogen peroxide significantly influenced the severity of the clinical presentation and the response to the therapy applied.

## Data availability statement

The original contributions presented in the study are included in the article/supplementary materials, further inquiries can be directed to the corresponding author/s.

## Ethics statement

The studies involving humans were approved by Mother and Child Health Institute of Serbia. The studies were conducted in accordance with the local legislation and institutional requirements. Written informed consent for participation in this study was provided by the participants’ legal guardians/next of kin.

## Author contributions

All authors have been active participants in the research (including participation in the conception, execution, and writing of the manuscript). Authors confirm: that the work described has not been published before; that it is not under consideration for publication anywhere else; that its publication has been approved by all co-authors, as well as by the responsible authorities.
